# Immunological Mechanisms for Hepatocellular Carcinoma Risk after Direct-Acting Antiviral Treatment of Hepatitis C Virus Infection

**DOI:** 10.3390/jcm10020221

**Published:** 2021-01-10

**Authors:** Pil Soo Sung, Eui-Cheol Shin

**Affiliations:** 1 Division of Gastroenterology and Hepatology, Department of Internal Medicine, College of Medicine, Seoul St. Mary’s Hospital, The Catholic University of Korea, Seoul 06591, Korea; 2 The Catholic Liver Research Center, The Catholic University of Korea, Seoul 06591, Korea; 3 Laboratory of Immunology and Infectious Diseases, Graduate School of Medical Science and Engineering, Korea Advanced Institute of Science and Technology (KAIST), Daejeon 34141, Korea; 4 The Center for Epidemic Preparedness, KAIST Institute, Daejeon 34141, Korea

**Keywords:** hepatitis C virus, hepatocellular carcinoma, direct-acting antivirals

## Abstract

Direct-acting antiviral agents (DAAs) that allow for rapid clearance of hepatitis C virus (HCV) may evoke immunological changes. Some cases of rapid de novo hepatocellular carcinoma (HCC) development or early recurrence of HCC after DAA treatment have been reported. During chronic HCV infection, natural killer (NK) cells exhibited a deviant functional phenotype with decreased production of antiviral cytokines and increased cytotoxicity; however, DAA treatment rapidly decreased their cytotoxic function. Effective DAA therapy also suppressed the intrahepatic activation of macrophages/monocytes. This was followed by a decrease in mucosal-associated invariant T (MAIT) cell cytotoxicity without normalization of cytokine production. Rapid changes in the phenotypes of NK and MAIT cells after DAA treatment may attenuate the cytotoxicity of these cells against cancer cells. Moreover, DAA treatment did not normalize the increased frequencies of regulatory T cells even after clearance of HCV infection. Thus, the persistently increased frequency of regulatory T cells may contribute to a local immunosuppressive milieu and hamper the clearance of cancer cells. This review will focus on recent studies describing the changes in innate and adaptive immune responses after DAA treatment in patients with chronic HCV infection in the context of de novo occurrence or recurrence of HCC.

## 1. Introduction

Approximately 71 million individuals are infected with hepatitis C virus (HCV) worldwide, and acute HCV infections frequently result in chronic, long-term infections [1]. Since its discovery, significant advances have been made in HCV diagnosis and treatment [1]. However, hepatocellular carcinoma (HCC) is highly likely to develop in cases of chronic hepatitis C (CHC) due to the direct oncogenic effect of viral proteins and the indirect oncogenic effect of chronic inflammation, fibrogenesis, and dysfunctional immunity [2]. Globally, HCC is the sixth most common malignancy and the third most common cause of malignancy-related deaths [1].

Pegylated interferon-alpha (peg-IFN-α) and ribavirin have been used as a therapeutic combination for chronic HCV infection. Newer direct-acting antivirals (DAAs) targeting non-structural viral proteins are better tolerated by patients and result in a markedly increased rate of sustained virological response (SVR) [3]. This significant breakthrough has been reinforced by the use of pan-genotypic agents. These agents also have a favorable side effect profile, and their administration periods are shorter than those of their traditional counterparts. Despite a nearly 100% cure rate, DAA therapy does not prevent HCV reinfection [4].

The immune cell population within the liver comprises both innate and adaptive immune cell types, such as natural killer (NK) and natural killer T (NKT) cells, and B and T lymphocytes [5]. Interestingly, several significant differences have been observed between the murine liver immune cell population and their human counterparts. A higher proportion of NK cells are generally found in human than in murine liver, although the frequencies of dendritic cells (DCs) and Kupffer cell populations are similar in mouse and human livers [6]. In addition, mucosal invariant T (MAIT) cells are present at a higher frequency in the human liver, whereas NKT cells are present in greater numbers in the murine liver [6]. Chronic HCV infection causes alterations in the frequency, function, and phenotype of these immune cells [7]. However, whether the innate and adaptive immune systems, affected by several years of constant antigenic stimulation and endogenous interferon (IFN) production, are restored to normal function after elimination of HCV by DAAs has not been completely elucidated. In the last few years, studies have shown that exhausted HCV-specific CD8^+^ T cells fail to recover entirely, although DAAs rapidly eliminate HCV from the whole body [8,9]. Thus, when the patient is re-exposed to HCV, there seems to be a deficient, memory-like adaptive response.

Patients with HCV-induced liver cirrhosis (LC) who achieved SVR by IFN-based treatment presented a lower risk of HCC development than those without treatment [10]. Since this study was published in 1995, further trials including control groups of IFN-non-responders have verified this result, including the Hepatitis C Antiviral Long-term Treatment against Cirrhosis (HALT-C) trial [11] and a trial conducted by the Swedish Hepatitis Group [12]. The clinical introduction of DAA therapy for HCV is relatively recent; therefore, long-term data on patients receiving DAAs are lacking. In very recent reports with patients who showed a complete response to HCC treatment, DAA therapy was associated with a significantly longer overall survival [13,14,15,16]. In patients with chronic HCV and pre-existing LC, the incidence of HCC seems to be diminished, but not completely eliminated, by DAA treatment [17,18,19,20,21,22]. A recent consensus recommended clinical vigilance after DAA treatment to detect new tumors in patients with advanced hepatic fibrosis (F3-4) [1,23]. Moreover, a high risk of recurrence or de novo occurrence of HCC shortly after DAA treatment in patients with cirrhosis was noted in some case studies [17,18,24]. These results have evoked considerable controversy and discussion among international experts in this field. Recent meta-analyses have concluded minimal differences in the possibility of HCC development between the use of DAAs and IFN-based agents [25,26,27]. Compared to those treated with IFN-based regimens, a higher number of elderly patients with additional risk factors for HCC have been treated with DAAs. This discrepancy seems to be responsible for the higher incidence of HCC among patients treated with DAAs [25]. However, there is still a possibility that HCC may develop shortly after completion of DAA treatment due to the abrupt change in the immune microenvironment in the liver.

HCC is a typical example of a malignancy associated with chronic non-resolving inflammation [28,29,30]. Immune subtyping using data collected from The Cancer Genome Atlas (TCGA) describes HCC as a C4 subtype [31,32]. This subtype is associated with an enriched population of M2 macrophages and the suppression of Th1 CD4^+^ T cells. Moreover, NK cells are dysfunctional in the HCC tumor microenvironment [33]. These data indicate that the HCC microenvironment is largely controlled by immune cells that regulate and suppress antitumor immune responses [31,34]. When DAA therapy is administered without IFNs, the exclusive impact of HCV elimination on the immune system can be studied. In some patients, sudden alterations in the immune response may trigger two concerning events: hepatitis B virus (HBV) reactivation in subjects with HBV–HCV co-infection and HCC recurrence in individuals despite prior apparent clearance of the tumors [35].

This review focuses on recent studies describing changes in the innate and adaptive immune cell populations after DAA therapy for HCV infection in the context of HCC occurrence or recurrence. Each section of our review covers a single important player of intrahepatic innate and adaptive immunity: hepatocytes, NK cells, T cells (including γδ T cells, and MAIT cells), macrophages, and immune regulatory cells.

## 2. Possible Immunological Mechanisms of HCC Development following DAA-Induced Viral Clearance

The number of new cases of HCC seems to decrease when SVR is achieved by DAA therapy. However, the likelihood of HCC recurrence shortly after DAA administration remains controversial. A possible hypothesis to explain these findings is that HCV elimination with DAAs may influence the intrahepatic immune environment to create conditions that allow HCC tumorigenesis. The following sections will discuss the possible immunological mechanisms underlying HCC development following DAA-induced HCV clearance (Table 1).

### 2.1. Downregulation of IFN-Stimulated Genes in Hepatocytes after DAA Treatment

HCV-infected hepatocytes exhibit ongoing production of type I and III IFNs [7]. The influence of type I IFNs on tumor, immune, and endothelial cells through a range of pathways may delay the growth of tumors. Specifically, cancer progression may be slowed through the action of type I IFNs on malignant cells, leading to the cessation of the cell cycle, cell death, and augmented immunogenicity via the major histocompatibility complex (MHC)-1 molecule upregulation on the cell surface [36]. Furthermore, type I IFNs enhance pro-inflammatory cytokine release [36]. Cell cycle arrest and apoptosis are also induced through direct targeting of malignant cells by IFN-λs [37,38,39].

In the HCV-infected liver, type I and III IFNs produced in the infected liver are not sufficient to eliminate HCV infection. Moreover, HCV successfully replicates even when high IFN-stimulated gene (ISG) expression levels are sustained [40,41]. This upregulated expression of ISGs has been shown to negatively affect the outcome of peg-IFN-α and ribavirin treatments [7,41]. The IFNL4 genotype, which determines whether functionally active IFN-λ4 protein is produced, is the primary polymorphism explaining the unsuccessful responses to IFN-α-based therapy [7,40]. The allele that codes for the production of the fully active form of IFN-λ4, rs368234815-ΔG, is directly associated with ISG upregulation in the livers of HCV-infected patients [40]. Following DAA-induced HCV clearance, prompt ISG downregulation is seen in the liver. Using in vitro methods, our group recently demonstrated that HCV-infected primary human hepatocytes halted the production of IFN-λs, including IFN-λ4, after DAA treatment [42]. Even in cases in which the liver exhibited high ISG expression, DAA therapy rapidly downregulated the expression in patients for whom previous IFN treatment was ineffective [43,44,45,46]. A recent report elegantly demonstrated that HBV reactivation after DAA treatment is the result of attenuated hepatic type I and III IFN responses following HCV clearance in patients co-infected with HBV [47]. Treatment with DAAs also resulted in rapid normalization of the type I IFN response in peripheral blood mononuclear cells (PBMCs) based on ISG expression levels and STAT1 phosphorylation [46,48,49]. This downregulation of type I and III IFNs and ISGs in hepatocytes and immune cells, respectively, which include antitumor ISGs, may contribute to the occurrence or recurrence of HCC, similar to the reactivation of HBV replication.

### 2.2. Defective Tumor Surveillance by NK Cells after DAA Treatment

NK cells function as effectors through cytotoxic mechanisms and the production of cytokines [33,50]. In HCV infection, NK cells play a critical antiviral role in patients who are exposed to the virus at a subclinical level, such as intravenous drug users who are exposed to high HCV titers but do not become infected [51,52] or healthcare workers who are not infected with HCV despite a contaminated needle stick injury [53]. An elevated number of NK cells, together with augmented IFN-γ production and cytotoxicity, has been noted in such cases.

NK cells are also involved in the antitumor immune response by directly killing tumor cells. Early in hepatocarcinogenesis, deregulation of multiple immune-related genes associated with NK cell function was noted in a murine transgenic model (c-myc/tgfa) of an aggressive form of human liver cancer [33,54]. Notably, the frequency of NK cells in the peripheral blood of patients with HCC has been positively associated with recurrence-free survival [55]. Within the HCC microenvironment, the functions of NK cells are defective, which has been attributed to various causes [33,50,56]. Activating receptors, such as the NK group 2D (NKG2D), are critical for tumor immunosurveillance by NK cells. Major histocompatibility complex class I chain-associated molecules (MICs) A/B are recognized by NKG2D, and diminished MIC A/B expression has been associated with early hepatocarcinogenesis [33]. A previous study demonstrated that intratumoral NK cells exhibited NKG2D downregulation compared to NK cells in a non-tumor liver, resulting in the defective antitumor function of NK cells [57]. Rapid HCC development following DAA therapy has been postulated to be a consequence of the prompt downregulation of NKG2D-mediated immune surveillance by DAAs [58]. A previous study with 101 CHC patients treated with DAAs reported rapid recurrence of the tumors in 12 patients. A more abrupt decrease in NKG2D levels in NK cells following DAA treatment was associated with a higher risk of HCC recurrence in these patients [58,59].

**Figure 1:**
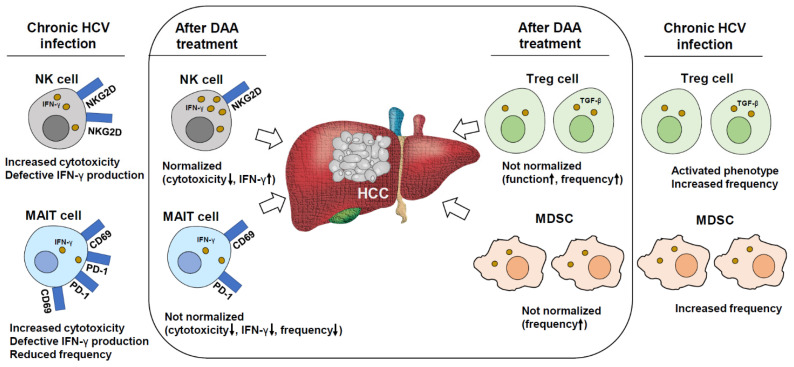
Changes in the phenotypes of immune cells after DAA-mediated HCV clearance. During chronic HCV infection, NK cells exhibited a deviant functional phenotype with decreased production of antiviral cytokines and increased cytotoxicity (represented by NKG2D upregulation). DAA treatment rapidly decreased their cytotoxic function. MAIT cells exhibited an activated phenotype with high expression of CD69, programmed cell death protein (PD)-1, and human leukocyte antigen (HLA)-DR. T-cell receptor-mediated stimulation of MAIT cells does not significantly contribute to the production of interferon (IFN)-γ in HCV-infected livers, resulting in defective overall IFN-γ production by MAIT cells. DAA treatment failed to recover the function of MAIT cells in response to T-cell receptor stimulation. An increase in the regulatory T (Treg) cell frequency is well-documented in chronic HCV infection. DAA treatment does not normalize the frequencies of regulatory T cells after clearance of HCV infection. The increased frequency of M-MDSC cells is not normalized 6 months after the end of DAA treatment.

In chronic HCV infection, IFN-α has been identified as an activator of intrahepatic NK cells [60]. Consequently, the function of NK cells is altered during chronic HCV infection, with amplified cytotoxicity and decreased levels of cytokine production, including IFN-γ (Figure 1) [61,62,63]. Thus, a functional dichotomy is created. After DAA treatment, the phenotype of NK cells changes rapidly. Golden-Mason et al. [64] observed decreased cytotoxicity of NK cells and downregulation of NKp30, NKp46, and tumor necrosis factor-related apoptosis-inducing ligand (TRAIL) after DAA treatment. Another study showed that NKp30 and NKp46 expression wee downregulated after DAA treatment [59,65]. Overall, these studies demonstrated the downregulation of NK cell cytotoxicity receptors and normalization of NK cell function following DAA treatment (Figure 1), which may paradoxically allow HCC development. Moreover, sustained defective NK cell diversity after DAA-mediated HCV clearance may also contribute to HCC development. Chronic HCV infection has been demonstrated to increase inter-individual, but decrease intra-individual, NK cell diversity. Furthermore, the defect in the NK cell repertoire diversity appears to be irreversible even after DAA treatment [66,67]. Thus, the global impact of HCV infection on the NK cell compartment may remain for years even though NK cell function (cytotoxicity or cytokine production) seems to be normalized with DAA treatment [66,67]. This impaired restoration of the NK cell repertoire may also be involved in hepatocarcinogenesis.

### 2.3. Sustained Impairment of T Cells after DAA Treatment

Immune cells need to penetrate the tumor tissue to exert antitumor effects. A higher number of infiltrated lymphocytes in the tumor correlates with reduced risk of HCC recurrence [55]. CD8^+^ T cells specific to tumor antigens are the main antitumor effector cells [68,69,70].

In chronic HCV infection, virus-specific T cells are exhausted and functionally impaired [7,71]. Whether the function of HCV-specific T cells is fully restored following achievement of SVR is a critical question. Peg-IFN-α-based therapies have been reported to not restore virus-specific T-cell function even after HCV is cleared [7]. An initial report regarding DAA treatment, demonstrated that it may partially reinvigorate exhausted virus-specific T cells [72]. Following DAA therapy, some degree of enhancement in the in vitro proliferation of HCV-specific CD8^+^ T cells was observed after peptide stimulation [72]. However, our group recently demonstrated that DAA-mediated viral clearance only transiently restores ex vivo virus-specific T-cell function [73]. This provides support to the theory that the exhausted phenotype of HCV-specific CD8^+^ T cells fails to recover after DAA therapy [74]. In chronic HCV infection, virus-specific CD8^+^ T cells are usually exhausted by persistent viral antigen stimulation [75]. These exhausted virus-specific T cells exist as two different subsets: TCF-1^+^CD127^+^PD-1^+^ memory-like T cells and PD-1^high^Eomes^high^CD127- terminally exhausted T cells [9]. Among these populations, the memory-like cells are maintained after DAA-mediated viral clearance and exhibit sustained impairment of functionality, unlike actual memory cells [9,76]. This persistent impaired functionality of memory-like HCV-specific CD8^+^ T cells may result in re-infection upon exposure to HCV after successful DAA treatment [77]. Another recent report demonstrated that HCV-specific CD8^+^ T cells remain functionally impaired after HCV clearance due to the constant, non-restorative mitochondrial dysfunction [8]. For CD4^+^ T cells, a DAA-mediated viral clearance does not reinvigorate exhausted CD4^+^ memory T-cells in chronic HCV infection [78]. Functional impairment of gamma delta T cells has also been shown to not be restored by DAA therapy [79]. Functional changes in tumor antigen-specific T cells after DAA treatment need to be investigated in HCV-infected patients with previously recovered HCC. If sustained functional impairment of HCV-specific T cells and gamma delta T cells is involved in the occurrence or recurrence of HCC after DAA treatment, this also needs to be examined.

Currently, the approved second-line treatments in HCC are the immune checkpoint inhibitors (ICIs) nivolumab and pembrolizumab, and atezolizumab (+ bevacizumab) is a first-line treatment for unresectable tumors. Nivolumab and pembrolizumab are anti-PD-1 while atezolizumab is an anti-PD-L1. In the adjuvant setting, remnant cancer cells invisible in imaging studies may cause early recurrence of HCC. An anti-PD-1/PD-L1 treatment may be promising in this case as these agents reinvigorate exhausted tumor-specific T cells and clear remnant cancer cells by immune-mediated cytotoxicity. In contrast, NK cells express minimal levels of the PD-1 molecule; therefore, anti-PD-1/PD-L1 treatment is not likely to have direct effects on NK cell function [80].

### 2.4. Changes in Macrophage-Derived Cytokines and Sustained Dysfunction of MAIT Cells after DAA Therapy

Chronic inflammation is accompanied by crosstalk between immune cells and is largely dependent on the secreted cytokines [56]. The HCV-core protein is a potent agonist of the NOD-, LRR-, and pyrin domain-containing protein 3 (NLRP3) inflammasome, resulting in the production of active interleukin-1 β (IL-1β) from intrahepatic activated macrophages [59,81]. Serum CD163 is a functional marker of activated macrophages, and its serum titers significantly correlate with the levels of aspartate transaminase [82,83]. After clearance of HCV by DAAs, serum CD163 levels quickly decrease and the inflammatory activity of intrahepatic macrophages is markedly attenuated [83]. Other reports demonstrated that HCV-induced disruption of t soluble inflammatory mediators does not completely normalize even after DAA-mediated viral clearance [84,85]. Debes et al. [85] assessed serum cytokines in 13 patients who developed HCC following DAA therapy and found that the serum levels of chemokine (C-X-C motif) ligand 9, IL-22, TRAIL, a proliferation-inducing ligand (APRIL), vascular endothelial growth factor (VEGF), IL-3, tumor necrosis factor-like weak inducer of apoptosis (TWEAK), stem cell factor (SCF), and IL-21 were elevated to higher levels in patients who developed HCC after DAA treatment than in those who did not develop tumors [85].

The liver contains a considerable number of MAIT cells, which are sensitive to intrahepatic cytokines and bacterial products that translocate from the gastrointestinal system. MAIT cells are characterized by the expression of an invariant T-cell receptor segment (Va7.2), and CD161, and these cells can be activated by type I IFNs, IL-12, IL-15, and IL-18 [86]. The peripheral and intrahepatic frequencies of MAIT cells have been reported to be lower in individuals with HCV than in healthy controls [86,87]. MAIT cells exhibit signs of chronic immune activation and resulting immune exhaustion in chronic HCV infection [88,89]. Activation markers such as CD69, PD-1, and HLA-DR are upregulated on MAIT cells from chronic HCV-infected patients. Expression of exhaustion markers such as PD-1, CTLA-4, and Tim-3 are also increased (Figure 1) [88,89]. The cytokine IL-18, which is produced by macrophages and Kupffer cells, promotes inflammation in HCV-infected livers [90]. IFN-γ production by MAIT cells, which relies on IL-18, and T-cell receptor-mediated stimulation of these cells, does not contribute significantly to the production of IFN-γ in HCV-infected livers, resulting in defective overall IFN-γ production by MAIT cells (Figure 1) [86]. The IFN-γ-producing function of MAIT cells may be more severely impaired in HCV-HIV co-infection due to the marked dysbiosis that features both infections [91].

DAA therapy has been associated with an immediate and fast reduction of serum IL-18 levels [92,93]. Accordingly, DAA therapy rapidly decreases intrahepatic inflammation and MAIT cell cytotoxicity [86]. Moreover, throughout the 12-weeks of DAA treatment, the MAIT cell response (IFN-γ production) to T-cell receptor-mediated stimulation was constantly weak (Figure 1) [86]. This failure of MAIT cell recovery in response to T-cell receptor stimulation is also observed in patients with HCV-HIV co-infected patients treated with DAAs [94]. The defective functional recovery of MAIT cells in response to T-cell receptor stimulation and declining cytotoxicity of MAIT cells with the reduction of intrahepatic cytokine levels may be associated with HCC development or recurrence after DAA treatment.

### 2.5. Sustained Immune Suppression by Regulatory Cells after DAA Treatment

CD4^+^CD25^+^FoxP3^+^ regulatory T (Treg) cells contribute to the amelioration of intrahepatic T-cell activities in chronic HCV infection [75]. An increase in the Treg cell frequency in both the liver and peripheral blood is well documented in chronic HCV infection (Figure 1) [7,95,96,97,98]. Moreover, the frequency of Treg cells in the blood positively correlates with viral load [98]. However, neither IFN-based treatment nor DAA treatment decreases the Treg frequency (Figure 1). Recent studies have demonstrated that the numbers and functions of Treg cells do not return to normal status after DAA administration, even when evaluated after a lengthy follow-up period following viral eradication [99,100,101]. Interestingly, a higher frequency of Treg cells was reported following DAA therapy than before treatment in individuals who developed HCV-associated overt cryoglobulinemia vasculitis [101]. Thus, it has been postulated that the influence of Treg cells on the immune response is persistent, despite the apparent HCV eradication [99]. Notably, an increase in the frequency of circulating Treg cells with increasing HCC stage has been observed and inversely correlated with the number of tumor-specific CD8^+^ T cells [102]. This indicates that the sustained increase in Treg frequency after DAA-mediated HCV treatment may contribute to HCC occurrence or recurrence.

In several types of tumors, tumor-infiltrating Treg cells express high levels of PD-1 molecule, and recent reports demonstrated that blocking PD-1 enhanced their suppressive function [103,104]. A previous report showed the high expression of PD-1 on liver-infiltrating Treg cells in HCV-infected liver [105,106]. In that report, blocking the interaction between PD-1 and PD-L1 with anti-PD-L1 enhanced the in vitro suppressive function of Treg cells isolated from HCV-infected livers [105]. Therefore, when Treg cells are constantly increased with PD-1 upregulation even after DAA-mediated HCV clearance, adjuvant anti-PD-1 treatment may paradoxically contribute to the recurrence of HCC.

Myeloid-derived suppressor cells (MDSCs) also block T-cell responses in many human diseases. The frequency of peripheral monocytic-MDSCs in HCV-infected patients is significantly increased compared to healthy controls, which may favor viral escape and disease progression in HCV infection (Figure 1) [107]. Moreover, the frequency of MDSCs, defined as CD14^-^HLA-DR^-^CD11b^+^ CD33^+^ cells, correlates with the frequency of Treg cells in HCC [55,108]. HCV RNA was reported to be undetectable in most patients after a few weeks of DAA treatment, and the frequency of M-MDSC cells did not normalize 6 months after the end of the treatment (Figure 1) [109]. These results suggest that a sustained increase in the frequency of MDSCs in HCV infection may contribute to the occurrence or recurrence of HCC. The increases in Treg cells, MDSCs, or dysfunction of MAIT cells may be sustained for more than several months and may contribute to the later HCC development whereas the changes in NK cell phenotypes seem to occur abruptly and may contribute to the early development of HCC.

## 3. Summary and Conclusions

Although DAAs are unavailable to a considerable number of patients with CHC in some developing countries, they are anticipated to change the grim prognosis of hepatic morbidities related to HCV infection and improve the clinical outcomes of patients. Unfortunately, despite attaining viral clearance, the risk of HCC appears to remain in patients with HCV-related cirrhosis. Moreover, some patients develop de novo HCC or recurrent tumors shortly after DAA treatment. Insights into changes in the phenotypes and crosstalk among diverse immune cells after rapid viral clearance by DAA treatment assist clinicians in screening for the occurrence or recurrence of HCC after DAA treatment.

## Figures and Tables

**Table 1:** Potential immunological mechanisms involved in hepatocellular carcinoma (HCC) development after direct-acting antivirals (DAA)-mediated hepatitis C virus (HCV) clearance.

		Chronic HCV Infection	After HCV Clearance by DAAs
NK cell	Phenotype	Activated (NKG2D upregulation)	Normalized
	Function	Increased cytotoxicity Defective IFN-γ production	Normalized
	Diversity	Reduced	Not normalized
MAIT cell	Phenotype	Activated	Normalized
	Function	Increased cytotoxicity Defective IFN-γ production	Not normalized
	Frequency	Reduced	Not normalized
Treg cell	Phenotype	Activated	Not normalized
	Frequency	Increased	Not normalized
MDSC	Frequency	Increased	Not normalized

MAIT cell, mucosal-associated invariant T cell; MDSC, myeloid-derived suppressor cell; NK cell, natural killer cell; NKG2D, NK group 2D.

## Data Availability

Not applicable.
